# Nomogram for Predicting Depression Improvement after Deep Brain Stimulation for Parkinson’s Disease

**DOI:** 10.3390/brainsci12070841

**Published:** 2022-06-28

**Authors:** Bowen Chang, Chen Ni, Jiaming Mei, Chi Xiong, Peng Chen, Manli Jiang, Chaoshi Niu

**Affiliations:** 1 Department of Neurosurgery, The First Affiliated Hospital of USTC, Division of Life Sciences and Medicine, University of Science and Technology of China, Hefei 230001, China; changbowen21@163.com (B.C.); chenni_edu@126.com (C.N.); doctormeijiaming@163.com (J.M.); xc0624010052@126.com (C.X.); 15055163720@163.com (P.C.); dyjiangmanli@126.com (M.J.); 2 Anhui Province Key Laboratory of Brain Function and Brain Disease, Hefei 230001, China

**Keywords:** deep brain stimulation, Parkinson’s disease, depression, non-motor symptoms, nomogram

## Abstract

Background: Parkinson’s disease is a common neurodegenerative disease, with depression being a common non-motor symptom. Bilateral subthalamic nucleus deep brain stimulation is an effective method for the treatment of Parkinson’s disease. Thus, this study aimed to establish a nomogram of the possibility of achieving a better depression improvement rate after subthalamic nucleus deep brain stimulation in patients with Parkinson’s disease. Methods: We retrospectively analyzed 103 patients with Parkinson’s disease who underwent subthalamic nucleus deep brain stimulation and were followed up for the improvement of their Hamilton Depression scale scores 1 year postoperatively. Univariate and multivariate logistic regression analyses were used to select factors affecting the improvement rate of depression. A nomogram was then developed to predict the possibility of achieving better depression improvement. Furthermore, the discrimination and fitting performance was evaluated using a calibration diagram, receiver operating characteristics, and decision curve analysis. Results: The mean and median improvement rates of Hamilton Depression scores were 13.1 and 33.3%, respectively. Among the 103 patients, 70.8% had an improved depression, 23.3% had a worsened depression, and 5.8% remained unchanged. Logistic multivariate regression analysis showed that age, preoperative Parkinson’s Disease Questionnaire, Hamilton Anxiety, and Hamilton Depression scores were independent factors for the possibility of achieving a better depression improvement rate. Based on these results, a nomogram model was developed. The nomogram had a C-index of 0.78 (95% confidence interval: 0.69–0.87) and an area under the receiver operating characteristics of 0.78 (95% confidence interval: 0.69–0.87). The calibration plot and decision curve analysis further demonstrated goodness-of-fit between the nomogram predictions and actual observations. Conclusion: We developed a nomogram to predict the possibility of achieving good depression improvement 1 year after subthalamic nucleus deep brain stimulation in patients with Parkinson’s disease, which showed a certain value in judging the expected depression improvement of these patients.

## 1. Introduction

Parkinson’s disease (PD) usually occurs in individuals aged 55–65 years, affecting 1–2% of those aged over 60 years and approximately 0.3% of the total population, with a higher prevalence in men than in women [1,2]. The main motor symptoms in such patients include resting tremors, bradykinesia, and rigidity. Patients may also have a variety of non-motor symptoms (NMS), including mood disorders (e.g., depression, anxiety), cognitive disorders (e.g., frontal lobe dysfunction, memory difficulties, dementia), sleep disorders (e.g., apnea, sleep disorders), and autonomic dysfunctions (e.g., sexual dysfunction, sleep disorders, constipation) [3]. To address movement disorders, deep brain stimulation (DBS) was developed by Professor Benabid and has been used for more than 30 years since it was first applied in 1987 [4]. DBS has gradually become one of the most effective treatments for advanced PD, with numerous studies showing significant improvements in the motor symptoms of patients with PD, although controversy remains on its ability to improve depression [5,6,7]. Similarly, there is a lack of clinical models to predict the improvement of depressed mood in patients with PD who underwent DBS. The development of a nomogram cognitive map can fill this gap, as it integrates several important factors and predicts whether these patients can achieve better improvement in their depressed mood after DBS. Therefore, in this study, a retrospective study of patients with PD who underwent bilateral thalamic nucleus-DBS (STN-DBS) was conducted in our center with the aim of establishing and validating a nomogram to predict whether they would achieve better depressive improvement after 1 year.

## 2. Materials and Methods

### 2.1. Patients

Medical records and questionnaire results were retrospectively collected from patients with PD who underwent STN-DBS at the First Hospital of the University of Science and Technology of China from September 2019 to April 2021. The study protocol was approved by the ethics committee of our hospital (2021-N(A)-118). The patients included were middle-to-advanced PD patients, and the exclusion criteria were: moderate/severe cognitive impairment, persistent severe mental disorders, brain MRI showing severe atrophy or diffuse ischemic lesions, and systemic comorbidities that impeded surgery.

### 2.2. Outcome Assessment

Demographic and clinical variables, including age, sex, disease duration, and levodopa equivalent dose (LED), were collected from the patients’ medical records and questionnaires. Patients were evaluated with detailed scales to assess symptom severity, cognitive status, psychological status, and quality of life. The Unified Parkinson’s Disease Rating Scale (UPDRS-III) was used to assess symptom severity, whereas the Parkinson’s Non-Motor Symptom Scale (NMSS) was used to assess Parkinson’s non-motor symptoms. The Parkinson’s Disease Questionnaire (PDQ-39) scale was used to evaluate quality of life, and the Hamilton Anxiety (HAMA) and Hamilton Depression (HAMD) scales were used to assess psychological status. Cognitive status was also assessed using the Montreal Cognitive Assessment (MoCA) and Simple Mental State Examination (MMSE) scales. One year postoperatively, the patients’ depressive status was reassessed using the HAMD scale. Patients were then divided into two groups based on their median improvement rate 1 year postoperatively, classifying those above the median improvement rate as the high improvement rate group and those below the median improvement rate as the low improvement rate group.

### 2.3. Statistical Analyses

The Empower(R) (www.empowerstats.com, accessed on 1 June 2022, X&Y solutions, Inc Boston, MA, USA) and R (http://www.R-project.org, accessed on 1 June 2022) software was used for all statistical analyses. Normal distribution of the variables was first examined through the Kolmogorov–Smirnov test. Normally distributed data were then evaluated using the two-tailed Student’s *t*-test or one-way analysis of variance (ANOVA), whereas non-parametric data were compared among different groups using the Mann–Whitney U test. Logistic regression multivariate analysis of variance was also used to explore related risk factors, which were then used to develop the regression model and transformed into a nomogram. Calibration plots, receiver operating characteristic (ROC) curve, and decision curve analysis (DCA) diagram were further used to assess the model. C-index * C-index is calculated by Hmisc package in R *. Lastly, correlations of HAMD score improvement rate with preoperative PDQ-39, HAMD scores and UPDRSIII improvement rate were analyzed using the Pearson’s correlation coefficient test.

## 3. Results

### 3.1. Patients

Final analysis was performed in 103 patients with PD (Table S1). Demographic data of all subjects are shown in Table A1. All patients were enrolled consecutively, and none of the 103 patients had been lost to follow-up. Patients were aged from 35–75 years, of which 63 (61.17%) were male and 40 (38.83%) were female. The mean and median improvement rates of HAMD score 1 year postoperatively was 13.1% and 33.3%, respectively, showing that STN-DBS significantly reduced patients’ HAMD scores compared to their preoperative scores. Specifically, 70.8% of patients had an improved depression status, 23.3% had a worsened depression status, and 5.8% remained unchanged. We then divided the patients into higher and lower improvement rate groups according to their median improvement rate.

**Figure A1:**
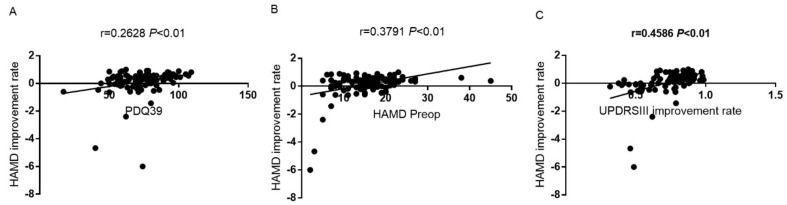
Correlations of HAMD score improvement rate with PDQ−39 (**A**), HAMD Preop (**B**) and UPDRSIII improvement rate (**C**).

The higher improvement rate group had 18 female cases (33.33%) with a mean age of 60.67 years. Meanwhile, the lower improvement rate group had 22 female cases (44.90%) with a mean age of 57.31 years. Differences in age, preoperative PDQ-39, MoCA, HAMA, and HAMD scores were statistically significant in both groups. On univariate analysis, correlations exist between the aforementioned parameters. Table A2 further details the univariate and multivariate regression analysis, age (OR = 1.08, 95% CI: 1.00–1.12, *p* = 0.0432), preoperative PDQ-39 (OR = 1.11, 95% CI: 1.04–1.19, *p* = 0.0012), HAMA (OR = 1.17, 95% CI: 1.02–1.34, *p* = 0.0281), and HAMD scores (OR = 1.10, 95% CI: 1.01–1.19, *p* = 0.0335); these were independent risk factors in the improvement of HAMD scores postoperatively in patients with PD. Table A3 further details the multivariate regression analysis for each variable in both groups. In addition, the preoperative PDQ-39, HAMD scores and UPDRSIII improvement rate in patients with PD were positively correlated with the improvement rate of HAMD scores 1 year postoperatively (Figure A1).

### 3.2. Development of the Nomogram

**Figure A2:**
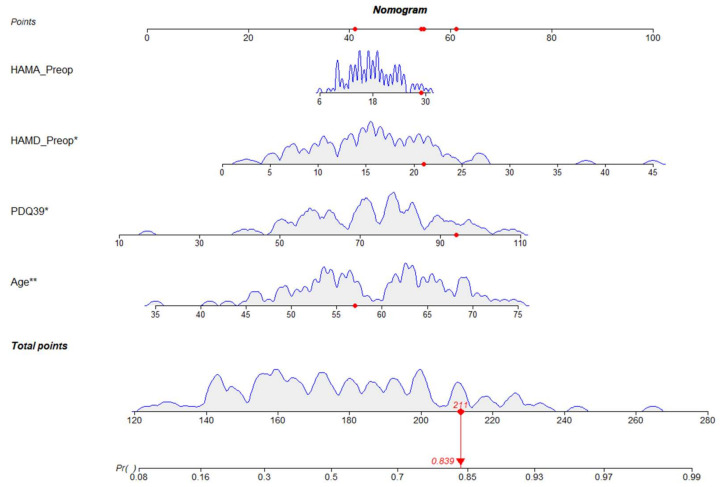
Nomogram to predict improvement of HAMD score after STN-DBS for PD. Clinical factor corresponds to a specific point by drawing a line straight upward to the points axis. After the sum of the points is located on the total points axis, the sum represents the probability of getting higher HAMD score improvement rate. * *p* < 0.01 ** *p* < 0.001.

**Figure A3:**
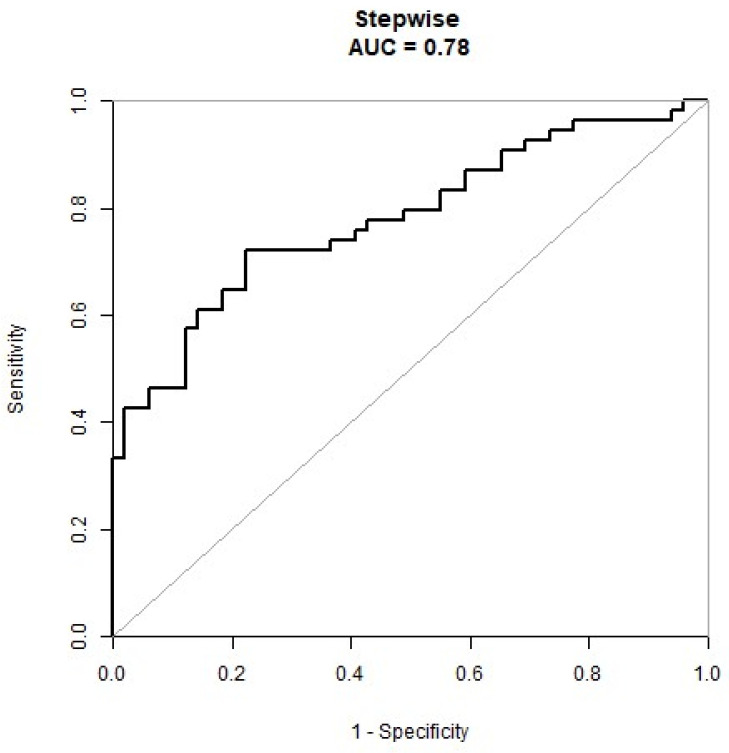
A receiver operating characteristic curve to evaluate the discriminating capability of the nomogram.

Based on these results, we developed a prediction model and generated a line graph to predict whether the HAMD score could be improved one year after STN-DBS (Figure A2). Each clinical factor corresponded to a specific score, and a linear point axis was plotted to calculate the total score, which corresponded to a higher probability of improving the HAMD score. As shown in Figure A3, the prediction model had good discriminant ability, and the area under the ROC curve was 0.78 (95% CI: 0.69–0.87).

### 3.3. Validation of the Nomogram

**Figure A4:**
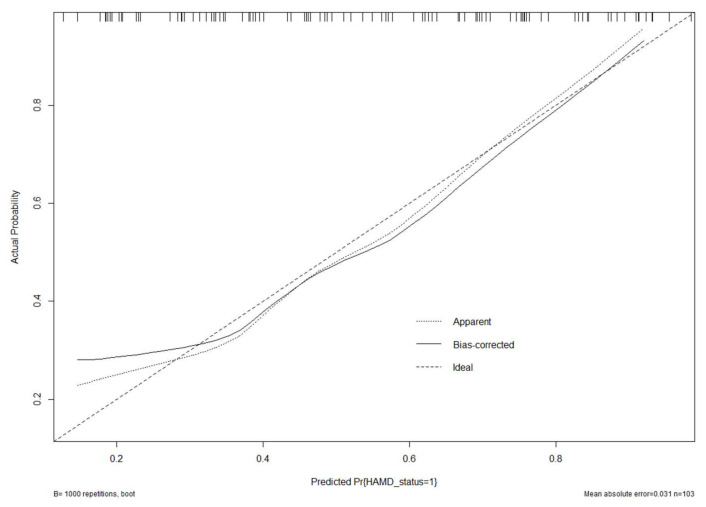
Calibration curve of the model. The calibration of the model in line with the agreement between predicted and observed outcomes of improvement of HAMD score.

**Figure A5:**
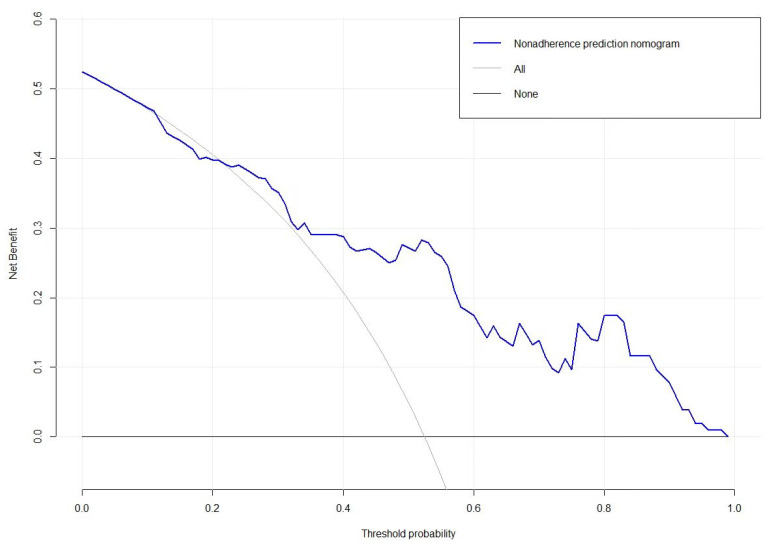
The decision curve analysis diagram of the model.

The generated model was internally verified using the bootstrap verification method, with a C-index of 0.78. Calibration curves were also generated by plotting the actual and predicted improvements in HAMD scores between the better HAMD score improvements (*Y*-axis) and better HAMD score improvements (*X*-axis). Results in Figure A4 show good agreement between the predictions and observations. Additionally, the DCA was drawn with the net benefit rate as the ordinate and the high-risk threshold as the abscissa (Figure A5), with the high-risk threshold set to (0.27, 0.9).

## 4. Discussion

Depression is a common neuropsychiatric complication of PD, occurring in approximately 20–30% of patients [8]. As DBS has gradually become the main treatment method for middle and advanced PD, the influence of DBS on depressive symptoms has aroused a lot of attention. Several preliminary studies have shown the short-term effectiveness of DBS in the treatment of depression in PD [9]. Moreover, a meta-analysis that reviewed 82 studies on the behavioral outcomes of patients with PD who underwent STN-DBS found that 8% experienced depression postoperatively. Depression is usually relieved with appropriate psychotropic medication, which is why patients may still meet the criteria for depression after treatment albeit with significant reduction in symptoms [10,11,12,13,14,15]. In contrast, Castelli and colleagues found that depression in the STN-DBS group did not improve at the 3-year follow-up, as compared to the control group who received levodopa and did not undergo DBS [16].

In our study, we found that STN-DBS can effectively improve depression in patients with PD. One year postoperatively, depression was noted to have improved in 70.8% of patients, worsened in 23.3%, and remained unchanged in 5.8%. Therefore, for a more beneficial preoperative evaluation and to guide patient expectations, it is very important to explore which factors affect the benefit of depression improvement in patients after STN-DBS surgery. A previous study showed positive changes in depressive symptom outcomes after DBS, although these changes were not adjusted for other factors, such as quality of life or improved motor control [17]. Contrarily, the present study found that the improvement of patients’ depression 1 year postoperatively may be related to their age, preoperative PDQ-39, MoCA, HAMA, and HAMD scores. On multivariate logistic regression analysis, we found that the patients’ years of age, preoperative PDQ-39, HAMA, and HAMD scores were independent influencing factors in the improvement of their depression 1 year postoperatively. Age has been an important predictor of depression in numerous studies [18,19], which was consistent with our study showing that older patients were more likely to achieve satisfactory improvement. In addition, preoperative PDQ-39 and HAMD scores were positively correlated with the improvement rate and were found to be independent factors in affecting the improvement of depression. Patients with poorer preoperative quality of life and more severe depression also had a greater chance of improvement, possibly attaining a higher rate of improvement. Therefore, the quality of life and the depressive state of patients should be fully evaluated preoperatively to set reasonable expectations in the improvement of their depressive mood after treatment. Furthermore, the preoperative HAMA score was an independent factor in affecting the improvement of postoperative depression, suggesting that patients with a more severe preoperative anxiety were more likely to have an improved depressive mood after surgery.

* Given its clinical heterogeneity, the improvement in depression after STN-DBS may be multifactorial, associated with improved motor function, sleep quality, quality of life, and reduced medication in individual patients. In addition, studies have shown that STN-DBS stimulation may affect brain structures responsible for emotion. Specifically, STN has marginal regions, and transmission of stimulation to these regions may influence depression [20]. Stimulation may also affect other monoaminergic pathways that are important for regulating mood [21] *.

However, even if patients have the same risk factors, the likelihood of achieving better depression improvement is completely different. Poor improvement of depression can be caused by a variety of factors, and single-factor analysis may unilaterally affect the prognosis. As a result, other important factors are often missed, thereby creating inaccurate judgements on the prognosis of patients. Nomogram clinical prediction is a good tool to address these concerns. It predicts the prognosis of patients by integrating the influence of various factors, has been widely used in the survival analysis of patients with various diseases, and it has gradually replaced the traditional prediction model. Unfortunately, this predictive model is rarely used in patients with PD who underwent DBS. To the best of our knowledge, only two studies in 2019 have developed nomograms in these patients—XQ Wang’s nomogram based on postoperative delirium and Frizon LA’s nomogram on postoperative life quality improvement [22,23,24]. No other studies have been reported on cognitive improvement 1 year after STN-DBS. In our study, we incorporated 103 patients from 2017–2021 to construct the first nomogram model of postoperative depression improvement in patients with PD who underwent STN-DBS. Using multivariate analysis, four factors (age, preoperative PDQ-39, HAMA, and HAMD scores) were used as the nomogram score, resulting in a model with good predictive ability and a C-index of 0.78. In addition, the calibration graph in the validation queue was exactly the same.

Despite these findings, there are several deficiencies that should be noted in this study. * First, the study was a single-center design, so it’s not clear whether race, diet, climate or other factors affect a patient’s likelihood of getting a better depression improvement. * Moreover, the applicability of this model in primary hospitals or other fields has yet to be proven. To address this, we will adopt a multi-center cooperative approach and randomly select patients from other centers for external verification. In addition, this was a retrospective cohort study, making bias an inevitability in the follow-up process. Lastly, the follow-up time of this study is too short. In the later study, we will provide long-term follow-up results to explore the influence of STN-DBS on patients’ depression.

## 5. Conclusions

We have developed a nomogram to predict the possibility of achieving good depression improvement 1 year after STN-DBS in PD patients. In addition, the ROC curve, calibration plot, and DCA curve were used to show that the nomogram had a good predictive performance and calibration. This model has a certain value in judging the expected depression improvement of PD patients who underwent STN-DBS. * This can help doctors predict the state of postoperative depression, while it also has a certain reference for patients with surgical indications *.

## Data Availability

The original contributions presented in the study are included in the article/Supplementary Material; further inquiries can be directed to the corresponding author.

## Supplementary Materials

The following supporting information can be downloaded at: https://www.mdpi.com/article/10.3390/brainsci12070841/s1.

## Abbreviations

**LED**	levodopa equivalent dose
**UPDRSIII**	Unified Parkinson’s Disease Rating Scale III
**NMSS**	Parkinson’s Non-Motor Symptom Scale
**PDQ39**	Parkinson’s Disease Questionnaire 39
**MOCA**	The Montreal Cognitive Assessment
**MMSE**	Mini-Mental State Examination
**HAMD**	The Hamilton Depression Scale
**HAMA**	The Hamilton Anxiety Scale
**H-Y**	Hoehn-Yahr grading

## Appendix B

**Table A1:** Clinical data of all patients and comparison between patients with higher improvement rate of depression and those with lower rate of improvement.

	Total	Lower Improvement Rate	Higher Improvement Rate	*p*-Value
**No.**		49	54	
**Age (years)**	59.01 ± 8.03	56.59 ± 8.39	61.20 ± 7.06	0.003
**Education (years)**	5.54 ± 4.38	4.93 ± 3.89	6.09 ± 4.76	0.179
**LED**	659.95 ± 363.80	611.22 ± 375.25	704.17 ± 350.71	0.197
**Drug improvement rate**	0.52 ± 0.14	0.52 ± 0.13	0.52 ± 0.16	0.938
**UPDRSIII drug off**	54.00 ± 14.15	52.84 ± 12.51	55.06 ± 15.53	0.429
**UPDRSIII drug on**	26.64 ± 11.47	25.27 ± 10.13	27.89 ± 12.52	0.248
**NMSS Preop**	87.53 ± 30.01	82.14 ± 28.90	92.43 ± 30.42	0.082
**PDQ39 Preop**	73.24 ± 16.25	66.92 ± 14.62	78.98 ± 15.61	<0.001
**MOCA Preop**	18.67 ± 6.15	20.10 ± 6.04	17.37 ± 6.02	0.024
**MMSE Preop**	24.99 ± 3.74	25.55 ± 3.33	24.48 ± 4.04	0.148
**HAMD Preop**	15.73 ± 6.66	13.71 ± 5.12	17.56 ± 7.39	0.003
**HAMA Preop**	18.06 ± 5.38	16.51 ± 4.88	19.46 ± 5.47	0.005
**Duration (years)**	8.70 ± 3.80	8.20 ± 3.87	9.15 ± 3.71	0.209
**Sex**				0.229
male	63 (61.17%)	27 (55.10%)	36 (66.67%)	
female	40 (38.83%)	22 (44.90%)	18 (33.33%)	
**H-Y**				0.017
2	2 (1.94%)	2 (4.08%)	0 (0.00%)	
2.5	16 (15.53%)	9 (18.37%)	7 (12.96%)	
3	50 (48.54%)	29 (59.18%)	21 (38.89%)	
4	28 (27.18%)	8 (16.33%)	20 (37.04%)	
5	7 (6.80%)	1 (2.04%)	6 (11.11%)	

**Table A2:** Univariable and multivariate regression analysis showing the effect of the various factors on the improvement rate of depression.

Exposure	Univariable	Multivariable
	**OR 95%CI *p*-value**	**OR 95%CI *p*-value**
**Age (years)**	1.08 (1.02, 1.14) 0.0047	1.08 (1.00, 1.16) 0.0432
**Education (years)**	1.06 (0.97, 1.17) 0.1796	1.08 (0.93, 1.26) 0.3035
**LED**	1.00 (1.00, 1.00) 0.2034	1.00 (1.00, 1.00) 0.6882
**Drug improvement rate**	1.12 (0.07, 17.09) 0.9374	23.94 (0.06, 10315.49) 0.3049
**UPDRSIII drug off**	1.01 (0.98, 1.04) 0.4258	0.93 (0.85, 1.01) 0.0777
**UPDRSIII drug on**	1.02 (0.99, 1.06) 0.2467	1.07 (0.95, 1.20) 0.2703
**NMSS Preop**	1.01 (1.00, 1.03) 0.0857	0.95 (0.92, 0.99) 0.0054
**PDQ39 Preop**	1.06 (1.02, 1.09) 0.0004	1.11 (1.04, 1.19) 0.0012
**MOCA Preop**	0.92 (0.83, 1.03) 0.1487	0.91 (0.75, 1.10) 0.3182
**MMSE Preop**	0.93 (0.87, 0.99) 0.0262	1.00 (0.88, 1.14) 0.9914
**HAMD Preop**	1.12 (1.03, 1.21) 0.0069	1.17 (1.02, 1.34) 0.0281
**HAMA Preop**	1.11 (1.03, 1.19) 0.0052	1.07 (1.01, 1.19) 0.0335
**Duration (years)**	1.07 (0.96, 1.19) 0.2097	1.06 (0.91, 1.23) 0.4424
**Sex**		
male	1.0	1.0
female	0.61 (0.28, 1.36) 0.2304	0.20 (0.06, 1.71) 0.2328
**H-Y**		
2	1.0	1.0
2.5	4478298.90 (0.00, Inf) 0.9881	2414460.89 (0.00, Inf) 0.9930
3	4169450.70 (0.00, Inf) 0.9882	2109909.14 (0.00, Inf) 0.9931
4	inf. (0.00, Inf) 0.9872	2173729.34 (0.00, Inf) 0.9931
5	inf. (0.00, Inf) 0.9865	inf. (0.00, Inf) 0.9922

**Table A3:** Multivariate regression showing the effect of age, PDQ-39 Preop, MoCA Preop, HAMA Preop and HAMD Preop on the improvement rate of depression.

	Non-Adjusted		Model I		Model II	
	**OR (95% CI)**	***p*-Value**	**OR (95% CI)**	***p*-Value**	**OR (95% CI)**	***p*-Value**
**Age (years)**	1.08 (1.02, 1.14)	0.0047	1.08 (1.02, 1.14)	0.0059	1.06 (1.00, 1.12)	0.0432
**PDQ-39 Preop**	1.06 (1.02, 1.09)	0.0004	1.06 (1.03, 1.09)	0.0004	1.04 (1.01, 1.08)	0.0106
**MoCA Preop**	0.93 (0.87, 0.99)	0.0262	0.92 (0.86, 1.00)	0.0426	0.94 (0.86, 1.02)	0.1110
**HAMA Preop**	1.11 (1.03, 1.19)	0.0052	1.11 (1.03, 1.20)	0.0075	1.09 (1.00, 1.18)	0.0440
**HAMD Preop**	1.12 (1.03, 1.21)	0.0069	1.13 (1.04, 1.23)	0.0046	1.10 (1.01, 1.20)	0.0335

Model I is adjusted for duration and gender, whereas Model II is adjusted for duration, gender, education, H-Y, LED, NMSS Preop. CI, confidence interval; OR, odds ratio.
